# Wnt traffic from endoplasmic reticulum to filopodia

**DOI:** 10.1371/journal.pone.0212711

**Published:** 2019-02-22

**Authors:** Naushad Moti, Jia Yu, Gaelle Boncompain, Franck Perez, David M. Virshup

**Affiliations:** 1 Program in Cancer and Stem Cell Biology, Duke-NUS Medical School, Singapore; 2 Institut Curie, PSL Research University, Sorbonne Université, CNRS UMR144 “Cell Biology and Cancer”, Paris, France; 3 Department of Pediatrics, Duke University, Durham, North Carolina, United States of America; Baylor College of Medicine, UNITED STATES

## Abstract

Wnts are a family of secreted palmitoleated glycoproteins that play key roles in cell to cell communication during development and regulate stem cell compartments in adults. Wnt receptors, downstream signaling cascades and target pathways have been extensively studied while less is known about how Wnts are secreted and move from producing cells to receiving cells. We used the synchronization system called Retention Using Selective Hook (RUSH) to study Wnt trafficking from endoplasmic reticulum to Golgi and then to plasma membrane and filopodia in real time. Inhibition of porcupine (PORCN) or knockout of Wntless (WLS) blocked Wnt exit from the ER. Wnt-containing vesicles paused at sub-cortical regions of the plasma membrane before exiting the cell. Wnt-containing vesicles were associated with filopodia extending to adjacent cells. These data visualize and confirm the role of WLS and PORCN in ER exit of Wnts and support the role of filopodia in Wnt signaling.

## Introduction

Wnt proteins are secreted morphogens that play an important role in a variety of biological processes ranging from embryonic development, proliferation, differentiation, adult tissue homeostasis and cancers [1–3]. Wnts bind to cell surface receptors to activate diverse signaling pathways, the best-studied of which leads to the stabilization of β-catenin and the activation of target gene expression. Less is known about how Wnts travel from one cell to engage receptors on neighboring cells [4–6].

Newly synthesized Wnts are targeted to the lumen of the endoplasmic reticulum (ER) by signal peptides. There, they are modified by addition of a single mono-unsaturated palmitoleate by the membrane bound O-acyl transferase PORCN (porcupine) [3,5,7,8]. This palmitoleation is required for the next step, binding to the integral membrane carrier protein Wntless (WLS) in the ER [3,9,10]. The Wnt-WLS complex then shuttles to the Golgi where additional posttranslational modifications of Wnt can occur [11]. From the Golgi, Wnts bound to WLS move to the plasma membrane and then on to a Wnt receiving cell [10,12,13].

Several contrasting models have been proposed to explain how Wnts travel from the producing cell to the receiving cell (reviewed in [6]). All models must explain how the hydrophobic palmitoleate moiety on Wnt is protected from an aqueous environment. Similarly, they must explain how Wnts can be transferred across basement membranes, e.g. in the intestine where Wnts produced in stromal cells regulate crypt base columnar cells [14,15]. The simplest model is diffusion, where Wnts transfer in a gradient dependent manner, perhaps interacting with soluble Wnt binding proteins or with glycoproteins on the cell surface [16]. Wnts have also been identified on exosomal vesicles in both Drosophila and mammalian systems [17–19]. Another means by which Wnt transportation occurs is through WLS-containing exosomes across synapses, demonstrated in *Drosophila* [17,20,21]. Finally, Wnts may travel on signaling filopodia, also called cytonemes [20,22–24]. Indeed, in zebrafish models, filopodia have been visualized transporting Wnt8A from one cell to another [18].

**Fig 1:**
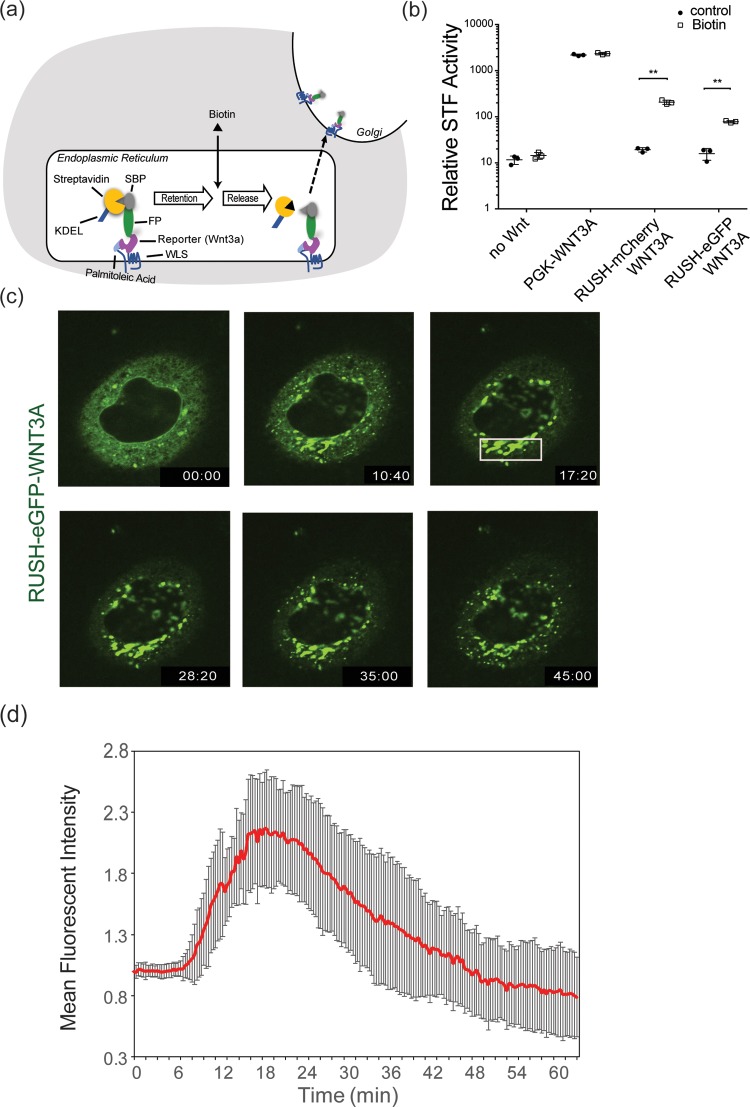
Synchronization of WNT3A protein using RUSH system. (a) Schematic of the RUSH-Wnt system. Under physiological conditions, KDEL -streptavidin binds to SBP-XFP-Wnt to retain the protein in the ER. Palmitoleated Wnts bind to WLS in the ER and their release is induced by the addition of 100 μM biotin. The SBP-XFP-Wnt is transported to the Golgi and subsequently to the plasma membrane. (b) RUSH-modified WNT3A is signaling-competent. β-catenin-dependent luciferase expression was stimulated by transient expression of the indicated proteins in SuperTopFlash cells. 100 μM biotin was added where indicated 5 hours after transfection, and luciferase activity was measured ~16 hours later (**p<0.01). (c) Fluorescence photomicrographs of HeLa cells expressing RUSH-eGFP-WNT3A at various time points after biotin addition (from S1 Video). At time 00:00 (minutes:seconds) of biotin addition, WNT3A remained in the ER. At time 10:40, WNT3A starts to leave ER and can be seen in the Golgi. At time 17:20, WNT3A is seen entirely in Golgi. Starting around 28:20 and continuing at 35:00 WNT3A can be seen in vesicle exiting the Golgi and moving towards the periphery. By time 45:00, the Golgi begins to be depleted of WNT3A. (d) Time-dependent Golgi localization of RUSH-eGFP-WNT3A. The plot shows fluorescence intensity in the Golgi region (white box in Fig 1C) at different time points after biotin addition. Intensities were normalized to the maximal Golgi intensity with arbitrary units. (n = 7 independent cells ± SD).

The variety of potential mechanisms suggests there is a need to develop new experimental approaches to visualize Wnts during secretion. Previous studies have shown that there are several routes for protein secretion and transport in the cells. To dissect the route that a particular cargo protein uses, specialized assays are needed that can be adaptable to a variety of cargo proteins present in a cell [25]. The temperature block assay that retains protein in the ER at 15°C and in the Golgi at 20°C [26,27], has been a powerful tool to study intracellular trafficking of specific proteins in living cells but these assays are limited in scope and ability to study cells in physiological conditions. A method that relies on retention-release mechanism of cargo molecules was developed recently [25]. The system, called Retention Using Selective Hook (RUSH), contains two components. The protein of interest is fused to the streptavidin-binding peptide (SBP) and a genetically encoded fluorescent reporter [25]. Second, streptavidin is fused to a protein stably located in the ER. This can be as simple as a KDEL sequence that serves as the ER hook. At steady state, streptavidin-KDEL will be bound to SBP and the cargo will be retained in the ER. Upon biotin addition, cargo proteins with their fluorescent tags will be released from the streptavidin-KDEL. Their subsequent trafficking can be visualized using real-time imaging in living cells [25] (Fig 1A).

Here, using RUSH, we followed the path of Wnt proteins from the ER to the membrane. The studies confirm that the RUSH-Wnt model is a useful system to study post-ER regulators of Wnt transport machinery. We find that both PORCN and WLS are required for Wnts to exit the ER, and provide new evidence for the involvement of filopodia in transmitting Wnt signals.

## Material and methods

### Cells and reagents

HeLa, HEK293T and RKO cells were cultured in Dulbecco's modified Eagle medium (DMEM) (Invitrogen) supplemented with 10% of Fetal Bovine Serum (FBS) (Hyclone), 1% sodium pyruvate (Lonza) and 1% penicillin and streptomycin (Invitrogen) (complete medium) at 37°C and 5% of CO_2_. HeLa and RKO cells were transfected using Lipofectamine 2000 Reagent (Invitrogen) using manufacturer’s protocol. Biotin was purchased from Sigma and used at a concentration of 100 μM. CellMask Deep Blue and Deep Green plasma membrane dyes were purchased from Invitrogen. The PORCN inhibitor ETC-159 was formulated in DMSO and used at a final concentration of 100 nM as previously described [28].

### Plasmids

The RUSH plasmid containing both streptavidin-KDEL as a hook and SBP-eGFP/mCherry-E-cadherin as a reporter has been described previously [25]. Briefly, E-cadherin was removed by restriction digestion using *FseI* and *SfiI* enzymes and replaced by human *WNT3A* and *WNT8A* [29] that were cloned by PCR. Plasmids Str-KDEL_SBP-mCherry-WNT3A and Str-KDEL_SBP-EGFP-WNT3A will be deposited at Addgene.

The STF reporter cell line (HEK293T cells with the firefly luciferase gene under the control of eight tandem repeats of TOPFlash TCF/LEF responsive promoter element) was a gift from Kang Zhang (University of California San Diego, La Jolla, CA) [30].

### CRISPR-Cas9

The WLS CRISPR knockout cells were generated by transient transfection of the pLentiCRISPRv2-Cas9 plasmid from the Zhang lab (Addgene plasmid #52961) [31]. The non-targeting control gRNA targeting sequences was GTTCGACTCGCGTGACCGTA, and the WLS gRNA targeting sequence for KO1 clone was AGGGGGGGCGCAAAAATGGC (in 5’-UTR of WLS exon 1) and TGCGCCGGGGGAATCCGTGC (in the 5’-UTR and ORF boundary) for KO2 clone. Briefly, RKO cells were transiently transfected with 2 μg of either non-targeting control or WLS gRNA construct in a 60-mm dish and selected for five days using puromycin. Limiting cell dilution was used to isolate single clones. The two knockout clones both had a large deletion in Exon 1 resulting in removal of the start codon. The expression of WLS in the two KO clones were verified by both qPCR and western blotting.

### Live cell imaging

HeLa cells were seeded onto Nunc Lab-Tek 4-well chambered cover glass with non-removable wells (ThermoFisher Scientific) one day prior to transfection. 24 hours after transfection with RUSH-WNT3A expression plasmids, the medium was removed and D-biotin (Sigma-Aldrich) at 100 μM final concentration was introduced in the chamber. Time-lapse acquisition and z-stack imaging was done at 37°C in a thermostat-controlled stage-top incubator using N-STORM with Andor CSU-W1 spinning disk confocal microscope (Nikon). Images were acquired using a 60x objective and NIS-Element Advanced Research software (Nikon). Raw images were processed using Imaris 9.0 (Bitplane, Oxford Instruments) and analysed using Fiji 2.0, an image processing package of ImageJ [32][33].

### STF Wnt signaling assay

Wnt/β-catenin signaling assays were performed as previously described in HEK293T STF cells with the addition of biotin where indicated [9]. GraphPad Prism 7.0 was used for data analysis and graphics.

### Flow cytometry

HeLa cells were seeded on 6-well plates and transfected with RUSH-eGFP-WNT3A plasmid. After 24 hours, 100 μM biotin was added with or without 100 nM ETC-159. The cells were collected at different time points by treating with non-enzyme cell dissociation buffer (Sigma). The detached cells were washed twice with ice-cold PBS + 10% FBS+ 1 mM EDTA and incubated with anti-GFP antibody conjugated with PerCP-eFluor710 (clone 5F12.4, eBioscience) for 60 min. The cells were washed twice with ice-cold PBS + 10% FBS+1mM EDTA and events were captured with BD LSR Fortessa (BD Biosciences). A total of 50,000 live cells were acquired and the data analyzed using FlowJo 10.

### Statistical analysis

Statistical analysis was performed using GraphPad Prism version 7.0 for Mac (GraphPad Software, La Jolla, CA) using one-way or two-way ANOVA, correcting for multiple comparisons using Tukey's test. Significance for all tests was set at *P*<0.05 unless otherwise stated.

## Results

### Synchronization of WNT3A protein secretion using the RUSH system

To study the Wnt secretory and transport mechanism, we used the RUSH (Retention Using Selective Hook) system [25] allowing us to analyze WNT3A localization in real-time (Fig 1A). We first tested if the RUSH-Wnt fusion constructs retained biological activity. RUSH-WNT3A constructs expressed in HEK293 cells with an integrated SuperTopFlash reporter [30] produced low levels of Wnt/β-catenin signaling. The addition of biotin to the culture medium increased signaling of two different RUSH-WNT3A constructs ~10-fold (Fig 1B), demonstrating that the fusion proteins were competent to signal, albeit 10-fold less active than unmodified WNT3A. We next followed the secretion of Wnt protein. We used HeLa cells because their morphology in culture made it easier to visualize the various cellular compartments. After expression in HeLa cells, RUSH-eGFP-WNT3A accumulated in the ER (Fig 1C and S1 Video). Following biotin addition, WNT3A was transported from ER to Golgi in a time dependent manner (S1 Video and Fig 1C and 1D). At t = 17:20 (min:sec), most of the WNT3A was transported to Golgi (Fig 1C and 1D). Wnt protein resided in the Golgi for approximately 20 minutes, presumably transiting from cis to trans-Golgi compartments and undergoing glycosylation. Subsequently, WNT3A was transported to the plasma membrane in small vesicles (Fig 1C). This pathway was not specific to WNT3A, as a RUSH-WNT8A construct showed similar results (S1 Fig and S2 Video).

### Wnt exit from ER requires palmitoleation and WLS

**Fig 2:**
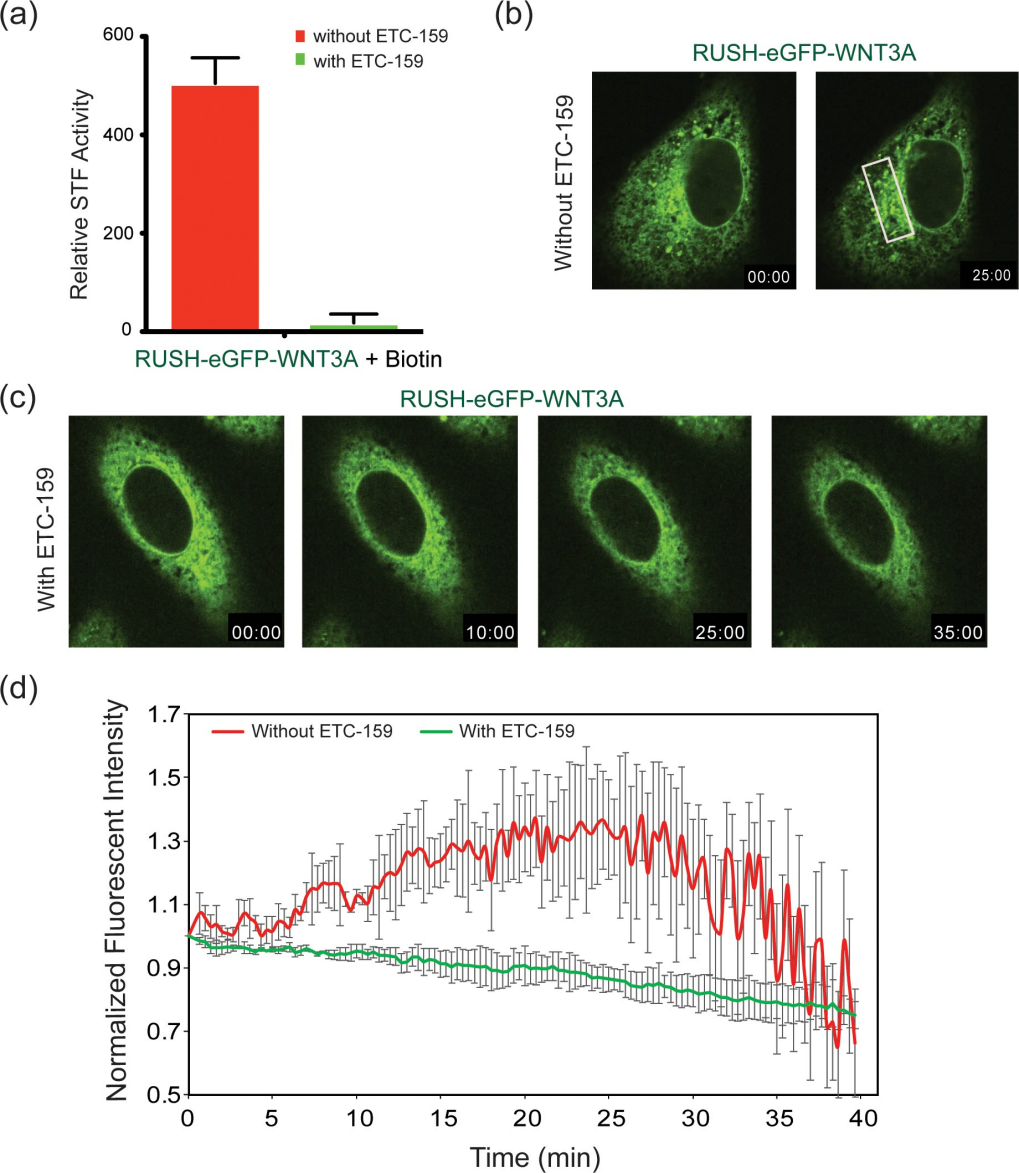
PORCN inhibition traps WNT3A in the ER. (a) SuperTopFlash assay in HEK293 cells transfected with RUSH-eGFP-WNT3A with and without ETC-159. 8–10 hrs post-transfection, cells were treated with and without 100 nM ETC-159 for 24 hrs. After 24 hrs, 100 μM biotin was added to induce the release of RUSH-eGFP-WNT3A from the ER. Luciferase readings were taken after 60 minutes biotin treatment. (b-c) Fluorescence photomicrographs showing time course of RUSH-eGFP-WNT3A trafficking in HeLa cells similar to Fig 1, except that 100 nM ETC-159 was added to the cells (in c) 18–24 hours prior to the addition of biotin where indicated. (d) Time-dependent analysis of Golgi localization of RUSH-eGFP-WNT3A in HeLa cells treated with and without 100 nM ETC-159, as in Fig 1. Intensities were normalized to the maximal Golgi intensities with arbitrary units (white box in Fig 2B).

To further assess if the RUSH-WNT3A constructs followed the known Wnt secretion pathway, we examined the effect of blocking PORCN or WLS activity on RUSH-eGFP-WNT3A secretion. PORCN palmitoleates Wnt proteins in the ER, and loss of PORCN activity blocks Wnt secretion and activity [28,34,35]. Inhibition of PORCN activity with the PORCN inhibitor ETC-159 abrogated signaling from the RUSH-eGFP-WNT3A fusion protein, confirming that the fusion protein behaves like native WNT proteins (Fig 2A). Live cell imaging showed that the same concentration (100 nM) of ETC-159 abolished WNT3A exit from the ER (Fig 2B and 2C and S3 Video). Time-dependent analysis of WNT3A movement to the Golgi (indicated by the white box in Fig 2D) confirms palmitoleation is required for WNT3A to exit the ER and move to the Golgi (Fig 2D).

**Fig 3:**
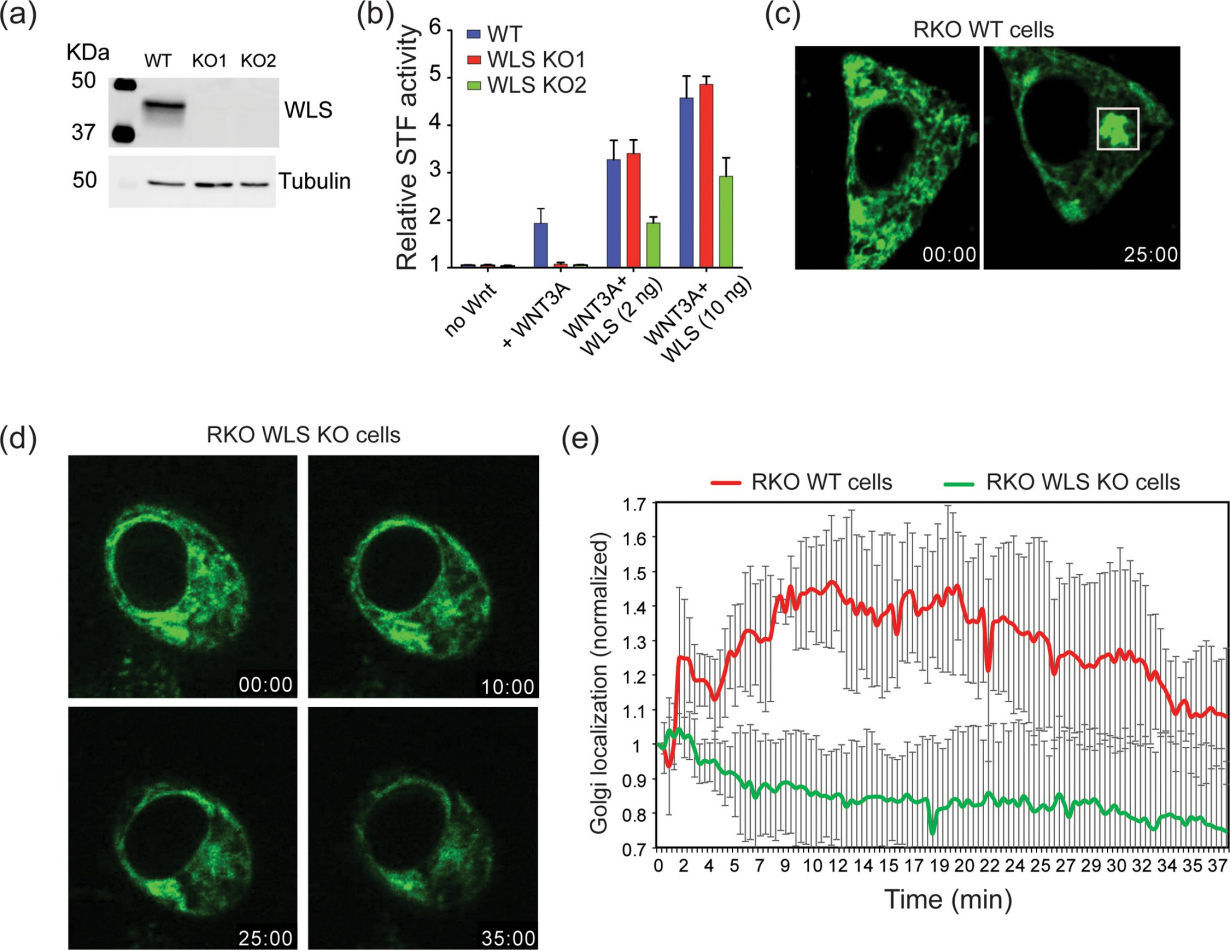
WLS knockout abolishes WNT3A trafficking. (a) Western blot analysis of WLS-depleted RKO cells. Cell lysates were assessed for WLS protein levels after CRISPR-Cas9 targeting and single cell cloning and tubulin was used as loading control. The blot shows two different CRISPR-Cas9 targeted clones. (b) SuperTopFlash assay in RKO wildtype and RKO WLS KO cells. The assay shows complete loss of Wnt signaling in RKO WLS KO cells and rescue by transfection with 2 ng or 10 ng wildtype WLS expression plasmid. (c) Fluorescence photomicrographs of RKO wildtype cells transfected with RUSH-eGFP-WNT3A illustrate transport from ER (time = 00:00) (minutes:seconds) to Golgi (time = 25:00) after biotin addition. (S4 Video) (d) Fluorescence photomicrographs of RKO WLS KO cells transfected with RUSH-eGFP-WNT3A showing WNT3A does not exit the ER upon biotin addition. (e) Time-dependent analysis of Golgi localization of RUSH-eGFP-WNT3A, analyzed as in Fig 1C and 1D, in wildtype and WLS KO RKO cells. (n = 4 cells per condition ± SD).

Following palmitoleation, Wnts bind to WLS in the ER, a step required for transport to the Golgi [3]. To further understand the role of WLS in Wnt trafficking and signaling, we employed the CRISPR-Cas9 system to knockout *WLS* in RKO cell lines that are normally WLS-high. Fig 3A shows that we achieved knockout of WLS at the protein level in two independent clones. Confirming the requirement for WLS, both knockout lines lost Wnt/β-catenin signaling and could be rescued by re-expression of wild type WLS (Fig 3B). Live cell imaging of cells transfected with RUSH-eGFP-WNT3A showed Wnt exit from the ER was blocked in RKO WLS KO cells (Fig 3C and 3D and S4 Video). Re-expression of WLS in the RKO WLS KO cells rescued the trafficking, allowing WNT3A to move out of the ER (S5 Video). These data illustrate the important roles of PORCN and WLS in WNT3A trafficking and further confirm the physiological nature of the RUSH-WNT3A construct.

### Wnt vesicles pause in sub-membranous region

**Fig 4:**
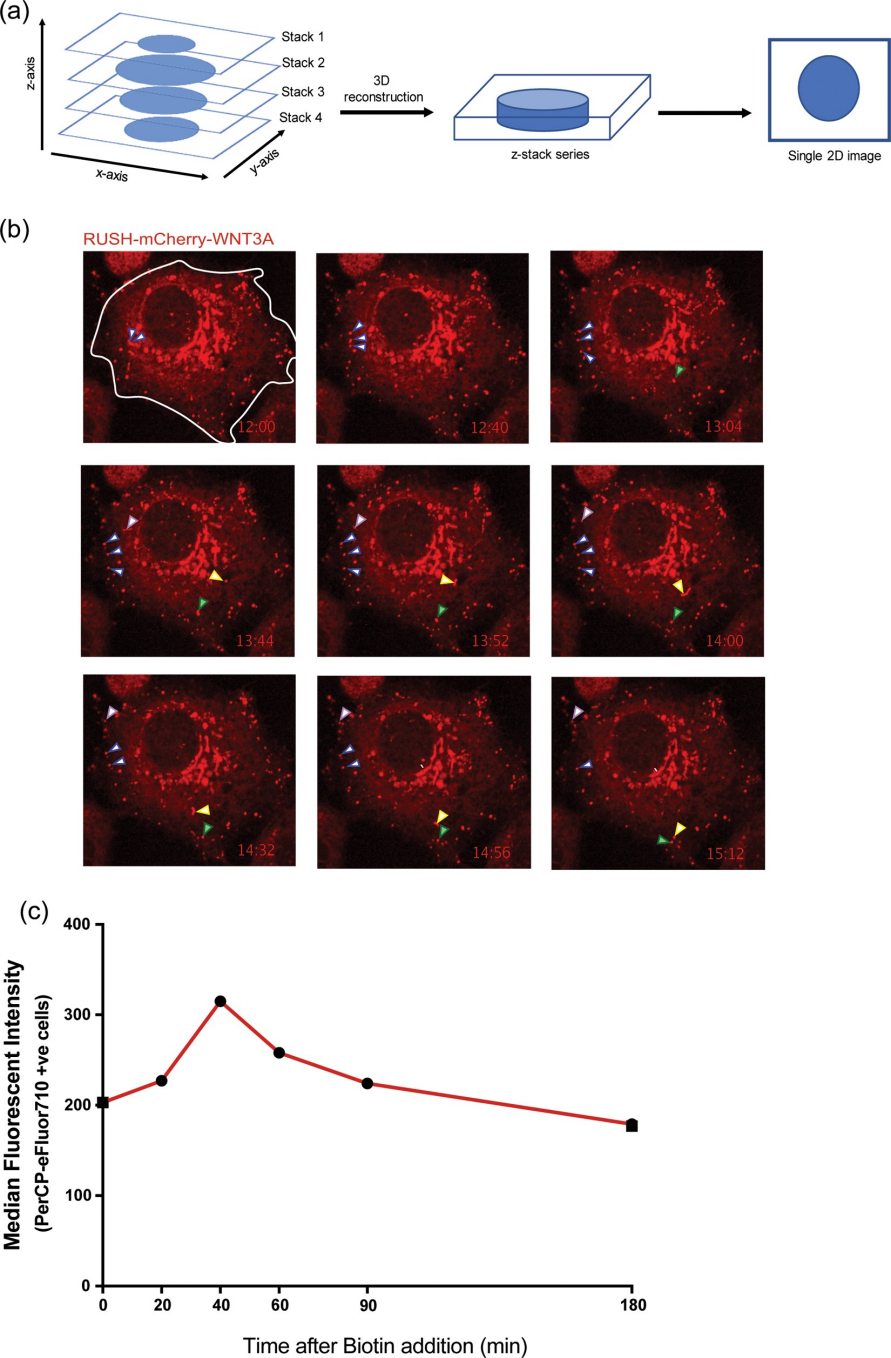
WNT3A time course to the membrane. (a). Multiple focal depth z-stack imaging was used to visualize vesicles leaving the Golgi after biotin addition. Different focal planes along the z-axis in a single cell were acquired at multiple time points after Wnt release. During analysis, each z-stack series was flattened to the xy-plane to generate the 2D images and movies. (b) Wnt vesicles pause in sub-plasma membrane regions. Individual flattened z-stacks images of HeLa cells expressing RUSH-mCherry-WNT3A. 18 hrs post-transfection, 100 μM biotin was added to the cells and time course z-stack imaging was started at t = 12:00 to visualize vesicular transport to the PM. The various arrows follow individual vesicles to illustrate their pause near the plasma membrane prior to presumed membrane fusion. (c) Wnt-containing vesicles fuse with the plasma membrane. HeLa cells expressing RUSH-eGFP-WNT3A were treated with 100 μM biotin at t = 0. Cell-surface Wnt was detected at the indicated time points by flow cytometry in non-permeabilized cells stained with anti-GFP antibody tagged with PerCP-eFluor710. Median fluorescent intensities of the PerCP-eFluor710 positive cells were used to plot the graph (representative of three independent experiments).

We followed the path of Wnt from Golgi to plasma membrane. Time-lapse z-stack imaging of HeLa cells expressing RUSH-mCherry-WNT3A revealed WNT3A vesicles pausing immediately below the plasma membrane (PM) for up to several minutes before disappearing, presumably by membrane fusion events although these were not directly visualized (Fig 4A and 4B and S6 Video). We also did not observe re-uptake of RUSH-mCherry-WNT3A by endocytosis.

We next examined the fate of RUSH-GFP-WNT3A after the vesicles reached the cell surface (Fig 4C). To test if the RUSH-Wnt protein was indeed exposed on the cell surface, we used an antibody against GFP and performed flow cytometry in non-permeabilized cells at various time points after biotin addition. GFP-WNT3A was detectable at the cell surface at steady state, and increased 20–40 minutes after biotin addition, indicating transport of the GFP-WNT3A to the cell surface. This wave of biotin-released GFP-WNT3A disappeared from the cell surface over the next hour, presumably by release or re-endocytosis. The study was repeated four times with similar results. Thus, the GFP-Wnt protein molecules do not appear to be retained on the cell surface for long periods.

### Wnts transfer from cell to cell via actin-based membrane extensions

**Fig 5:**
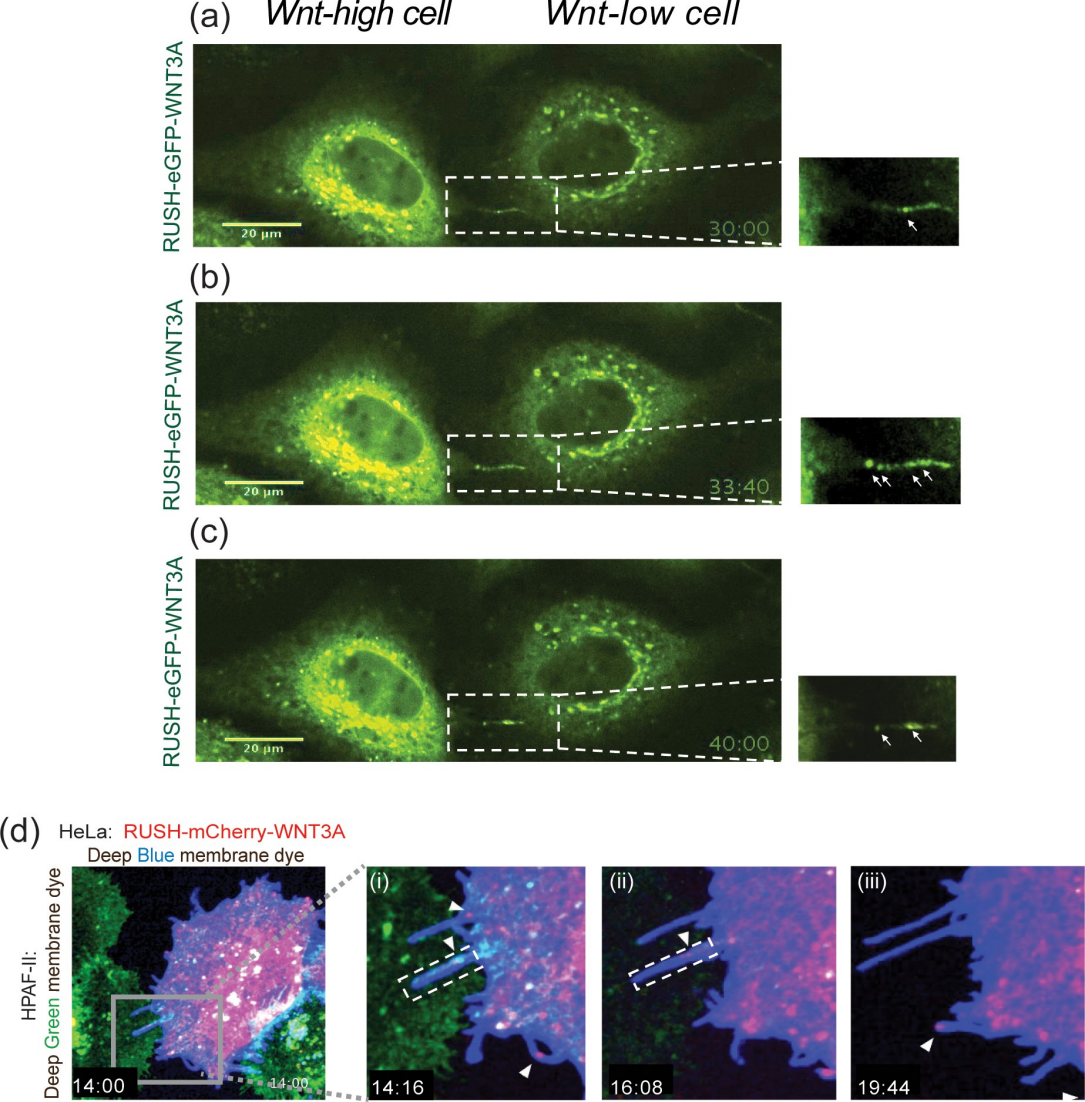
WNT3A transfer via filopodia. (a-c) Fluorescence photomicrographs of adjacent HeLa cells expressing RUSH-eGFP-WNT3A at indicated time points after biotin addition. From S7 video. (a) WNT3A is in the Golgi after biotin treatment. Inset showing WNT3A movement along filopodia from one WNT3A-high cell to another WNT3A-low cell (based on GFP intensities). Arrow shows filopodic bridge transporting WNT3A. (b) At t = 33:40, multiple vesicles are seen on the filopodia. (c) At t = 40:00. (d) Fluorescence photomicrographs of HeLa cells (expressing RUSH-mCherry-WNT3A and stained with CellMask Deep Blue membrane dye co-plated with RNF43-mutant HPAF-II cells stained with CellMask Deep Green membrane dye. From S9 Video (i) Inset showing filopodia extending from HeLa cell to HPAF-II cell at 14:16. (ii-iii) Inset showing WNT3A (mCherry) on the filopodia from HeLa cells moving to HPAF-II cells at indicated time points.

Membrane extensions or protrusions, including tunneling nanotubes, filopodia and signaling filopodia (also called cytonemes), are important in signal transduction, cell migration, environmental sensing, and in generating mechanical forces. They also play a role in trafficking of membrane-bound molecules and signaling vesicles [36–38]. Recent studies on Wnt trafficking have shown that WNT8A can be transported on cytonemes from one cell to another [18,39]. We used the RUSH-WNT3A model to further study Wnt trafficking via these membrane extensions. In a number of experiments, we were able to visualize RUSH-Wnt vesicles associated with long narrow cellular extensions. In Fig 5A, adjacent HeLa cells expressing RUSH-eGFP-WNT3A appeared to pass Wnt from one cell towards another via a ~1 um bridge, consistent with Wnt vesicle transport via a filopodia or tunneling nanotube (S7 and S8 Videos). Further studies are needed to determine if the Wnt is secreted near the receiving cell or transferred directly from one cell to the next.

Next, we co-plated RUSH-mCherry-WNT3A expressing HeLa cells (marked with blue membrane dye) with HPAF-II cells (labeled with green membrane dye) as another approach to visualize Wnt vesicle. HPAF-II cells have inactivating RNF43 mutations and hence have up-regulated cell surface Wnt receptors. Upon biotin addition, RUSH-mCherry-WNT3A from HeLa cells transported towards the target cells by cellular extensions resembling filopodia (Fig 5D and S9 Video). The extensions were typically between 1–6 μm in length although our system was not optimized to visualize longer extensions in multiple planes. Time-course z-stack confocal imaging revealed that RUSH-mCherry-WNT3A can be seen transporting from HeLa cells towards HPAF-II cells efficiently as compared to when HeLa cells plated alone (S9 Video). These data suggest that WNT3A are transported from one cell towards another via membrane protrusions resembling filopodia.

## Discussion

Cell-to-cell communication is a vital step in the regulation of growth, development and homeostasis via secretory proteins. Understanding the mechanism underlying the transmission of signals through tissues is of profound interest and requires robust assays for their study. We show here that the quantitative and real-time RUSH system is well suited to study the production and movement of biologically active fluorescent-tagged Wnt proteins from the time of their initial synthesis to their movement to the cell surface [40]. The RUSH system also demonstrated Wnt-containing vesicles travelling via filopodia to neighboring cells, a phenomenon previously described in Drosophila and zebrafish but not to our knowledge in human cells.

Initial studies on WLS showed it was a carrier for Wnts, but because those studies used a carboxyl-terminal epitope tag that masked the WLS ER localization signal, they mistakenly concluded that Wnt first binds to WLS in the Golgi. Subsequent work concluded that WLS binds Wnts in the ER, and here we confirm that in the absence of WLS, Wnt cannot exit the ER. Confirming this, in the presence of a PORCN inhibitor Wnt that is not palmitoleated and hence cannot bind to WLS also cannot exit the ER. Thus, WLS appears to be the essential carrier of Wnts from the moment of its acylation to the plasma membrane, and likely, beyond [17,20,40].

One key finding here is the fate of Wnt-containing vesicles as they approach the plasma membrane. Live cell imaging shows these vesicles to pause near the plasma membrane prior to their disappearance. The data here support two fates for these vesicles–fusion with the membrane with release of Wnts to the outside of the cell, and more interestingly, passage via cellular extensions consistent with filopodia. These filopodia show active movement of Wnts, presumably in vesicles, towards neighboring cells. In other settings, these signaling filopodia are able to transmit signaling via signaling synapses. Further refinement of the RUSH system may facilitate the detection of Wnt signaling synapses in human cells and tissues. The concept of Wnt signaling synapses may be relevant in the mammalian intestine where Wnts are known to be transported across basement membranes, from the intestinal PDGFRα-expressing myofibroblasts to the stem cell compartment including the crypt base columnar (CBC) cells [41,42]. The presence of extensive filopodial networks underlying the intestinal basement membrane has been noted by others [15,43,44].

Previous studies show that the active Wnt signaling occurs at plasma membrane (PM). It is of interest to examine both the Wnt-producing and Wnt-receiving cells. Our data highlights an important demarcation between the two cell types. Our data suggest Wnts do not reside for long on the PM of Wnt-producing cell. The newly produced Wnt may be re-endocytosed or passed to the Wnt-receiving cell where it forms a signalosome and initiates a Wnt/ß-catenin dependent signaling event [45–47]. Our data suggests a method of WNT3A transport from Wnt-producing cells, where Wnt vesicles pause near sub-cellular junctions of PM. A subset of these vesicles either fuse with the PM, or are passed to Wnt-receiving cells via cellular extensions resembling actin-based filopodia. Our images suggest that these filopodia vary in length. It was observed that filopodia were produced only from Wnt-producing cells. HeLa cells with close by Wnt-receiving HAPF-II cells showed the longest filopodia with WNT3A on them (Fig 5D). There are instances where WNT3A-containing filopodia retracts back and we assume this is due to absence of a nearby target cell. These data are consistent with filopodia as a transport mechanism for WNT3A.

The RUSH-Wnt system is a useful model system to study Wnt transport. It is relatively straightforward to utilize in cultured cells. The RUSH-Wnt system may also be useful in high-content screening applications. Further refinement may allow the study of Wnt transport in more complex systems such as organoid cultures to provide insights into how Wnts move from stroma Wnt-producing cells to tissue stem cells.

## Supporting information

S1 Fig Synchronization of WNT8A protein using RUSH system. (a) Fluorescence photomicrographs of HeLa cells expressing RUSH-eGFP-WNT8A at various time points after biotin addition, from S2 VIdeo. At time 00:00 (minutes:seconds) of biotin addition, WNT8A remained in the ER. At time 10:00, WNT8A can be seen in the Golgi. At time 20:00, WNT8A is seen entirely in Golgi. Starting around 45:00, WNT8A can be seen in vesicle exiting the Golgi and moving towards PM. By time 54:00, the Golgi begins to be depleted of WNT8A.
(b) Time-dependent analysis of ER-Golgi localization of RUSH-WNT8A. The plot shows fluorescence intensity in the Golgi region (white box in Fig (a)) at different time point after biotin addition. Intensities were normalized to maximum Golgi intensity. (n = 5 cells).
(TIF)

S1 Video Real-time imaging of the synchronized trafficking of RUSH-eGFP-WNT3A (corresponds to Fig 1). HeLa cells were transfected to express KDEL-Streptavidin as a hook and SBP-eGFP-WNT3A as a reporter. After 18 h of expression, at time 00:00, 100 μM biotin was added to induce the release and monitored using Nikon’s spinning disk confocal microscope.
(MP4)

S2 Video Real-time imaging of the synchronized trafficking of RUSH-eGFP-WNT8A (corresponds to S1 Fig). HeLa cells were transfected to express KDEL-Streptavidin as a hook and SBP-eGFP-WNT8A as a reporter. After 18 h of expression, at time 00:00, 100 μM biotin was added to induce the release and monitored using Nikon’s spinning disk confocal microscope.
(MP4)

S3 Video Real-time imaging of the synchronized trafficking of RUSH-WNT3A in the presence and absence of known PORCN inhibitor, ETC-159 (corresponds to Fig 2). HeLa cells were transfected with RUSH-eGFP-WNT3A and after 6–7 h of transfection, treated with ETC-159. 100 μM biotin was added ~12 h later.
(MP4)

S4 Video Real-time imaging of the synchronized trafficking of RUSH-WNT3A in RKO WT and RKO WLS KO cells (corresponds to Fig 3). Cells were transfected with RUSH-eGFP-WNT3A plasmid and 100 μM biotin was added 18 h later.
(MOV)

S5 Video Real-time imaging of the synchronized trafficking of RUSH-WNT3A with and without exogenous WLS. RKO WLS KO cells were transfected with RUSH-mCherry-WNT3A plasmid and 100 μM biotin was added 18 h later.
(MP4)

S6 Video Real-time z-stack imaging of the synchronized trafficking of RUSH-WNT3A (corresponds to Fig 4). HeLa cells were transfected with RUSH-mCherry-WNT3A plasmid and after 18 h of expression, 100 μM biotin was added and monitored using Nikon’s spinning disk confocal microscope. Z-stacks were analysed and merged on Fiji 2.0. Image acquisition was started ~12 min after biotin addition to minimize photo bleaching.
(MOV)

S7 Video WNT3A transfer via filopodia. Real-time imaging of the synchronized trafficking of RUSH-WNT3A (corresponds to Fig 5A). HeLa cells were transfected with RUSH-eGFP-WNT3A plasmid and after 18 h of expression, 100 μM biotin was added and monitored using Nikon’s spinning disk confocal microscope.
(MOV)

S8 Video WNT3A transfer via filopodia. Real-time imaging of the synchronized trafficking of RUSH-WNT3A (corresponds to Fig 5A). HeLa cells were transfected with RUSH-eGFP-WNT3A plasmid and after 18 h of expression, 100 μM biotin was added and monitored using Nikon’s spinning disk confocal microscope.
(MP4)

S9 Video Co-culture of Wnt producing and Wnt receiving cells. Real-time imaging of the synchronized trafficking of RUSH-WNT3A (corresponds to Fig 5D). HeLa cells transfected with RUSH-WNT3A and stained with CellMask Deep Blue membrane dye was co-plated with HPAF-II cells stained with CellMask Deep Green membrane dye. After 18 h of expression, 100 μM biotin was added and monitored using Nikon’s spinning disk confocal microscope. Images were acquired ~12 minutes after biotin addition to minimize photobleaching.
(MP4)
