# Supplementary figures and images for: Wnt traffic from endoplasmic reticulum to filopodia
Source: PLoS One. 2019 Feb 22;14(2):e0212711. doi: 10.1371/journal.pone.0212711 (PMC6386245; doi:10.1371/journal.pone.0212711)

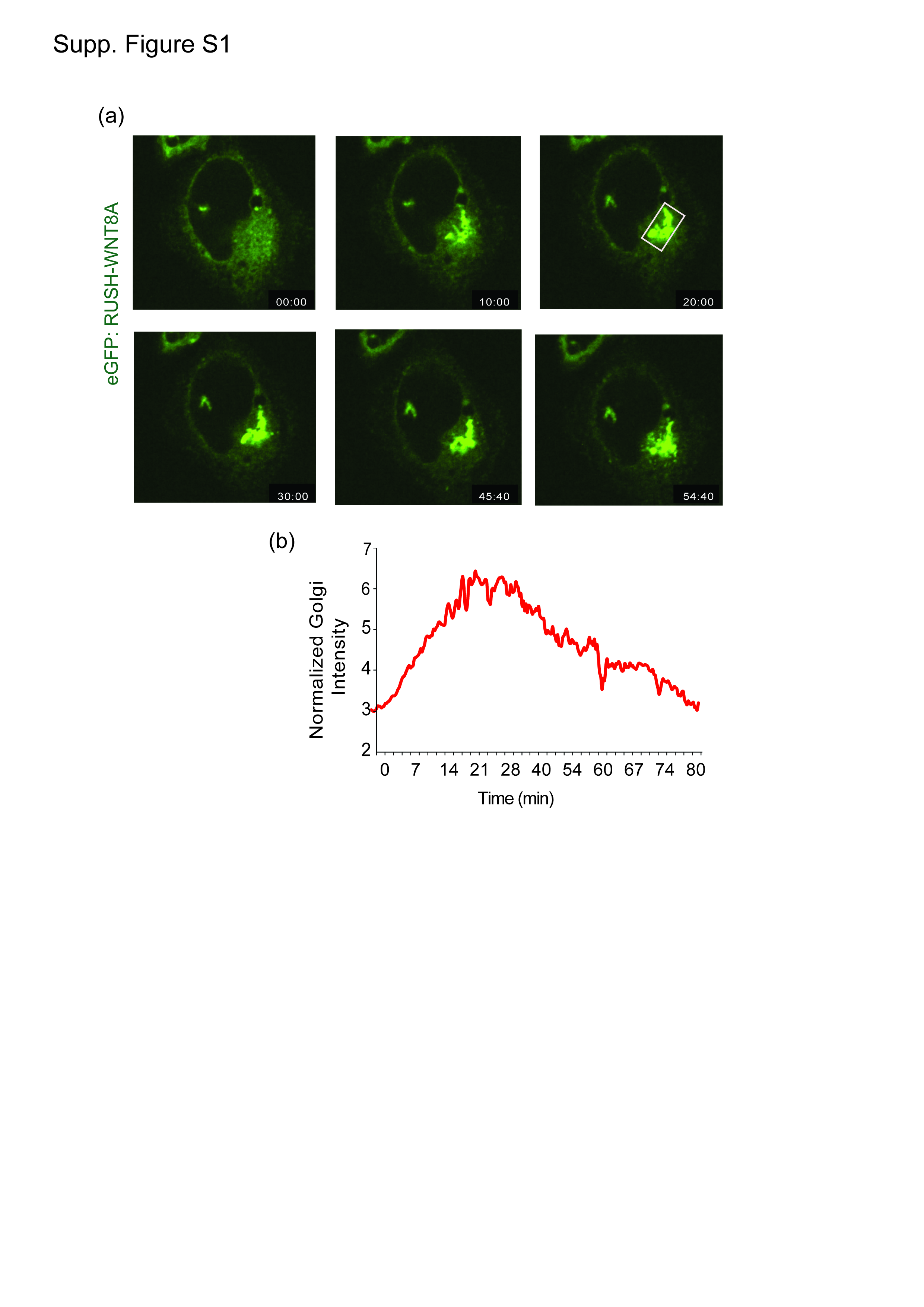

Supplement: S1 Fig — (a) Fluorescence photomicrographs of HeLa cells expressing RUSH-eGFP-WNT8A at various time points after biotin addition, from S2 VIdeo. At time 00:00 (minutes:seconds) of biotin addition, WNT8A remained in the ER. At time 10:00, WNT8A can be seen in the Golgi. At time 20:00, WNT8A is seen entirely in Golgi. Starting around 45:00, WNT8A can be seen in vesicle exiting the Golgi and moving towards PM. By time 54:00, the Golgi begins to be depleted of WNT8A. (b) Time-dependent analysis of ER-Golgi localization of RUSH-WNT8A. The plot shows fluorescence intensity in the Golgi region (white box in Fig (a)) at different time point after biotin addition. Intensities were normalized to maximum Golgi intensity. (n = 5 cells). (TIF) [file pone.0212711.s001.tif]
